# Molecular Characterization of Carbapenem-Resistant *Acinetobacter baumannii* Isolated from the Intensive Care Unit in a Tertiary Teaching Hospital in Malaysia

**DOI:** 10.3390/antibiotics10111340

**Published:** 2021-11-02

**Authors:** Jia Jie Woon, Cindy Shuan Ju Teh, Chun Wie Chong, Kartini Abdul Jabar, Sasheela Ponnampalavanar, Nuryana Idris

**Affiliations:** 1 Department of Medical Microbiology, Faculty of Medicine, University of Malaya, Kuala Lumpur 50603, Malaysia; jiajiewoon@siswa.um.edu.my (J.J.W.); cindysjteh@um.edu.my (C.S.J.T.); kartini.abduljabar@ummc.edu.my (K.A.J.); 2 School of Pharmacy, Monash University Malaysia, Subang Jaya 47500, Malaysia; 3 Department of Infectious Diseases, University of Malaya Medical Centre, Kuala Lumpur 50603, Malaysia; sheela@ummc.edu.my

**Keywords:** carbapenem-resistant *Acinetobacter baumannii*, intensive care unit, endemicity

## Abstract

The emergence of carbapenem-resistant *Acinetobacter baumannii* (CRAB) has now become a global sentinel event. CRAB infections often instigate severe clinical complications and are potentially fatal, especially for debilitated patients. The present study aimed to conduct molecular characterization on CRAB isolated from patients in the intensive care unit from 2015 to 2016 and determine the risk factors associated with patients’ mortality. One hundred CRAB isolates were retrospectively selected and included in this study. Antimicrobial susceptibility testing showed that all isolates remained susceptible to colistin, even though 62% of them conferred resistance to all other classes of antibiotics tested. OXA carbapenemase gene was found to be the predominant carbapenemase gene, with 99% of the isolates coharbouring *bla*_OXA-23_-like and *bla*_OXA-51_-like carbapenemase genes. All isolates were carrying intact *Car*O genes, with the presence of various degree of nucleotide insertion, deletion and substitution. Overall, PFGE subtyped the isolates into 13 distinct pulsotypes, with the presence of 2 predominant pulsotypes. Univariate analysis implied that age, infection/colonization by CRAB, ethnicity, comorbidity and CRAB specimen source were significantly associated with in-hospital mortality. Multivariate analysis identified a higher risk of mortality for patients who are of Chinese ethnicity with diabetes as an underlying disease. As CRAB infection could lead to high rate of mortality, comprehensive infection control measures are needed to minimize the spread of this pathogen.

## 1. Introduction

*Acinetobacter baumannii* has gained notoriety as one of the most prevalent nosocomial pathogens, causing substantial mortality and economic burden to hospitalized patients [1]. It is found predominantly in intensive care units (ICUs), affecting debilitated patients with compromised immunity, disrupted normal flora or barrier integrity [1]. It is known to be the causative agent for a wide range of infections such as skin and soft tissue infection, secondary meningitis, urinary tract infection, ventilator-associated pneumonia and bacteraemia, with crude mortality ranging from 20–60% [2,3,4].

Global surveillance estimated an incidence rate of *A. baumannii* infections at about 1,000,000 cases annually, of which more than half are associated with carbapenem resistance [5]. Undoubtedly, carbapenem-resistant *Acinetobacter baumannii* (CRAB) is now gaining global concern, to the extent that the World Health Organization has classified it as one of the critical priority pathogens that needs urgent research, discovery and development of new antibiotics [6]. Within six decades, since the 1970s, this bacterium has evolved from multidrug-resistant (MDR) to extensively drug-resistant (XDR) and finally established as pan-drug-resistant [7]. In large referral hospitals throughout the South and Southeast Asia region, most institutions register a carbapenem resistance rate of >50% [8]. In Malaysia, based on the National Surveillance of Antibiotic Resistance (NSAR), the rate of carbapenem resistance had risen from 49% in 2008 to 61% in 2016 and remained fairly constant at 60% yearly afterward [8].

Several mechanisms are found to contribute to the carbapenem resistance phenotype of this pathogen, including the production of carbapenemases, alteration of outer membrane porins and the influence of efflux pumps. Among these mechanisms, carbapenemases (carbapenem-hydrolysing β-lactamases), which belong to molecular class D OXA enzymes (oxacillinases), have emerged as the major mechanism responsible for carbapenem resistance in *A. baumannii* [9]. Class D carbapenemases such as *bla*OXA-23-like, *bla*OXA-24-like and *bla*OXA-58-like are responsible for the outbreaks in United States, Latin America, Europe, Asia and many parts of the world, with *bla*OXA-23-like being the most predominantly reported carbapenemase gene [2,10,11,12]. Among these carbapenemases, OXA-23 is a plasmid- or transposon-encoded gene, whereas OXA-51 is a chromosomal-based enzyme and is reported to be intrinsic to *A. baumannii*. OXA-24, on the other hand, can be plasmid-encoded or chromosomal-based, while OXA-58 is plasmid-encoded [2]. Besides the actions of enzymes, porins are also found to play a role in resistance to carbapenem. Porins such as *car*O and *opr*D constitute a channel for the influx of carbapenem. Thus, any form of alteration in these outer membrane porins, including the downregulation of *car*O porin system or the changes in the amino acid sequences, will reduce CRAB’s susceptibility to carbapenem by decreasing the antibiotic entry [2].

Due to its highly transmissible nature and remarkable environmental adaptive capacity, this superbug has contributed to countless outbreaks in ICUs [13]. Clonal assessment of this bacterium suggested that CRAB has become endemic in many ICUs, posing a serious threat to hospitalized patients who have underlying comorbidities [14,15]. The problem is exacerbated when the choice of antibiotic that is effective against infections caused by CRAB is increasingly compromised. Due to this reason, patients infected with CRAB often experience poor clinical outcomes such as prolonged hospital stay, significant morbidities and even death [16].

In Malaysia, phenotypic data on carbapenem resistance in *A. baumannii* have been well-documented by National Surveillance of Antimicrobial Resistance since 2003. Despite the consistency in monitoring the antibiotic resistance pattern, the available molecular and epidemiology data on CRAB remains rudimentary. Most studies in Malaysia have focused on comparative genomic characterization of a few strains of CRAB, and hence, comprehensive data related to its epidemiology are scarce [17,18]. This imposes a great challenge in managing and controlling the dissemination of CRAB in health care settings. In University of Malaya teaching centre (UMMC), the rate of CRAB has remained high in the general ICU at a rate of approximately 0.5 per 100 admission per year (unpublished hospital surveillance data), which is above the Malaysian national KPI for the incidence of multidrug-resistant *A. baumannii* of 0.3 per 100 admissions. Therefore, in this study, we aimed to characterize CRAB isolated from patients in the intensive care unit in a tertiary teaching hospital based on their molecular and phenotypic features. In addition, potential risk factors associated with patients’ in-hospital all-cause mortality were evaluated using statistical analysis as well.

## 2. Results

### 2.1. Overview of Patients with CRAB Cases

Complete clinical data for 89 out of 100 patients with CRAB isolated during ICU admission were available and included in the analysis. Among the 89 patients, 48 (53.9%) died during hospitalization, while the remaining 41 patients recovered and were discharged. Average length of hospitalization and ICU stay were 41.9 and 20.7 days, respectively. A total of 75.3% of the patients admitted to ICU had more than one comorbidity such as diabetes mellitus, hypertension and chronic kidney disease. Of these 89 patients, 57 of them were infected with CRAB, whereas the rest were colonized. Median duration for acquisition of CRAB in ICU was 9 days after admission to ICU.

The common empiric antibiotics prescribed to the 57 infected patients were imipenem (27 patients), meropenem (8 patients), ertapenem (2 patients), piperacillin–tazobactam (17 patients), ampicillin–sulbactam (4 patients), amoxicillin–clavulanate (1 patient), colistin (4 patients), trimethoprim–sulfamethoxazole (3 patients), doxycycline (1 patient) and beta-lactam antibiotics such as ampicillin (1 patient), ceftazidime (2 patients), cefepime (1 patient) and cefuroxime (1 patient). For targeted therapy, monotherapy with colistin, colistin + carbapenem and colistin + high dose ampicillin sulbactam were prescribed to 34, 1 and 9 patients, respectively. Within 3 days, 7 patients started targeted therapy. A total of 13 subjects succumbed to the infection prior to commencement of targeted therapy. The median duration to the starting of targeted therapy for the remaining 44 patients was 7 days.

Univariate analysis indicated that factors including older age, infection, Chinese ethnicity, presence of comorbidities, increase length of hospitalization and the administration of carbapenem as empiric treatment were significantly associated with patients’ in-hospital mortality. The details of patients’ clinical characteristics are summarized in Table 1.

### 2.2. Antimicrobial Susceptibility Profiles

**Figure 1:**
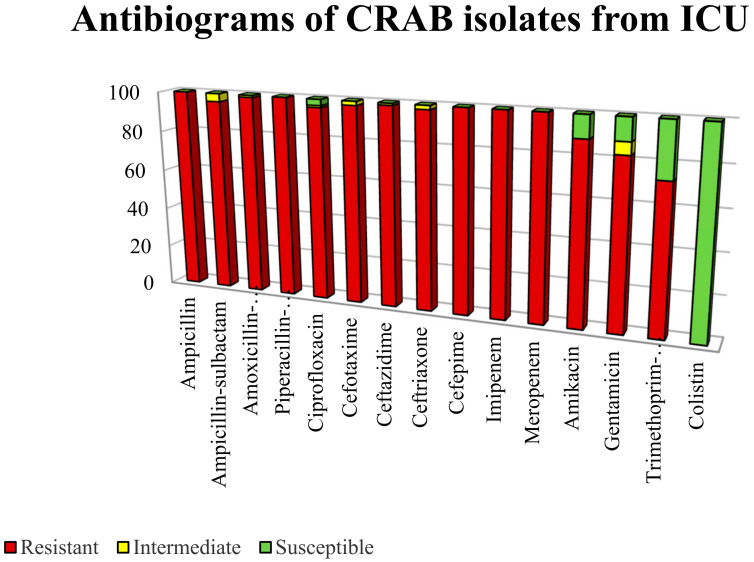
The antibiotic susceptibility profile of the CRAB isolated from intensive care unit.

Antimicrobial susceptibility profiles of CRAB isolates were determined based on AST-N314 card (BioMérieux, Marcy L’Etoile, France) using Vitek^®^ 2 system. A panel of antibiotics was tested, and the result is summarized in Figure 1. The result indicated that 100% of CRAB isolates showed nonsusceptibility to antibiotics from class beta-lactam, beta-lactamase inhibitor, cephalosporin, fluroquinolone and carbapenem. A total of 11% of the isolates remained susceptible to amikacin and gentamicin, whereas 27% of the isolates were susceptible to trimethoprim–sulfamethoxazole. None of the isolates showed resistance to colistin. In terms of minimum inhibitory concentration of imipenem, meropenem and colistin, the MIC ranges 16–128 µg/mL, 16–128 µg/mL and 0.5–2 µg/mL, respectively, were recorded.

### 2.3. Screening of Carbapenemase and Extended-Spectrum β-Lactamases (ESBL) Genes

In the present study, detection of commonly found carbapenemase and ESBL genes was performed, as described earlier. All the isolates harboured carbapenemase genes, with 99% of the isolates showing the coharbouring of *bla*OXA-23-like and *bla*OXA-51-like genes. The remaining one isolate was found to carry only *bla*OXA-51-like genes. The data suggested that carbapenem-hydrolysing Class D lactamase (CHDL) genes were the predominant carbapenemase genes found in our clinical setting. Class B metallo-β-lactamase genes were not detected in this study. In terms of ESBL genes screening, *bla*TEM was present in 83% of the isolates, while *bla*CTX-M and *bla*SHV remained negative for all isolates.

### 2.4. Carbapenem-Associated Outer Membrane Protein (CarO) Gene Analysis

*Car*O gene was present in all isolates. The analysis of 100 *Car*O porin nucleotide sequences revealed a product of 750 bp. During the analysis, there is no tandem repeat observed in the sequence, and all the isolates showed roughly the same number of amino acids, suggesting that no insertion sequence (IS) was present in the porin. Multiple sequence alignment with the *Car*O sequence of reference strains (ATCC 19606 and A6) allowed the categorization of these proteins into two major groups, which are *Car*Oa and *Car*Ob. *Car*Oa protein consisted of those that shared high similarity with carbapenem- susceptible reference strain ATCC 19606, while *Car*Ob protein comprised those which are highly similar to carbapenem-resistant A6 strains. Deduced amino acids comparison showed that 94% of the isolates were categorized into *Car*Ob, while the remaining 6% were grouped into *Car*Oa. Sequence comparison between carbapenem-resistant isolates in this study with the carbapenem-susceptible strain ATCC 19606 revealed the presence of multiple nucleotide substitutions, insertion and deletion along the nucleotide sequence. Nonetheless, there are six carbapenem-resistant isolates showing similar *Car*O amino acid sequence with ATCC 19606.

### 2.5. Genetic Relatedness of CRAB Isolates

**Figure 2:**
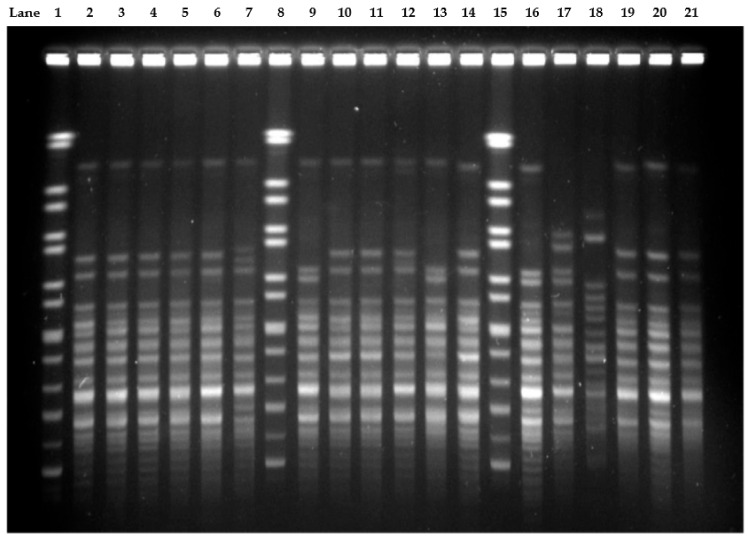
A representative PFGE gel image showing several CRAB pulsotypes in this study. M represents the marker used as a reference for generating the dendrogram. Lane 1, 8, 15: Marker; Lane 2: isolate 1601-5 (Ab4); Lane 3: isolate 1601-11 (Ab4); Lane 4: isolate 1601-16 (Ab4); Lane 5: isolate 1601-20 (Ab4); Lane 6: isolate 1602-9 (Ab4); Lane 7: 1602-12 (Ab5); Lane 9: isolate 1602-15 (Ab6), Lane 10: isolate 1603-6 (Ab4); Lane 11: isolate 1603-9 (Ab4); Lane 12: isolate 1603-11 (Ab4); Lane 13: isolate 1603-18 (Ab6); Lane 14: isolate 1604-1 (Ab4); Lane 16: isolate 1604-11 (Ab6); Lane 17: isolate 1604-12 (Ab1); Lane 18: isolate 1606-3 (Ab11); Lane 19: isolate 1606-5 (Ab4); Lane 20: isolate 1606-9 (Ab4); Lane 21: isolate 1508-18 (Ab4).

**Figure 3:**
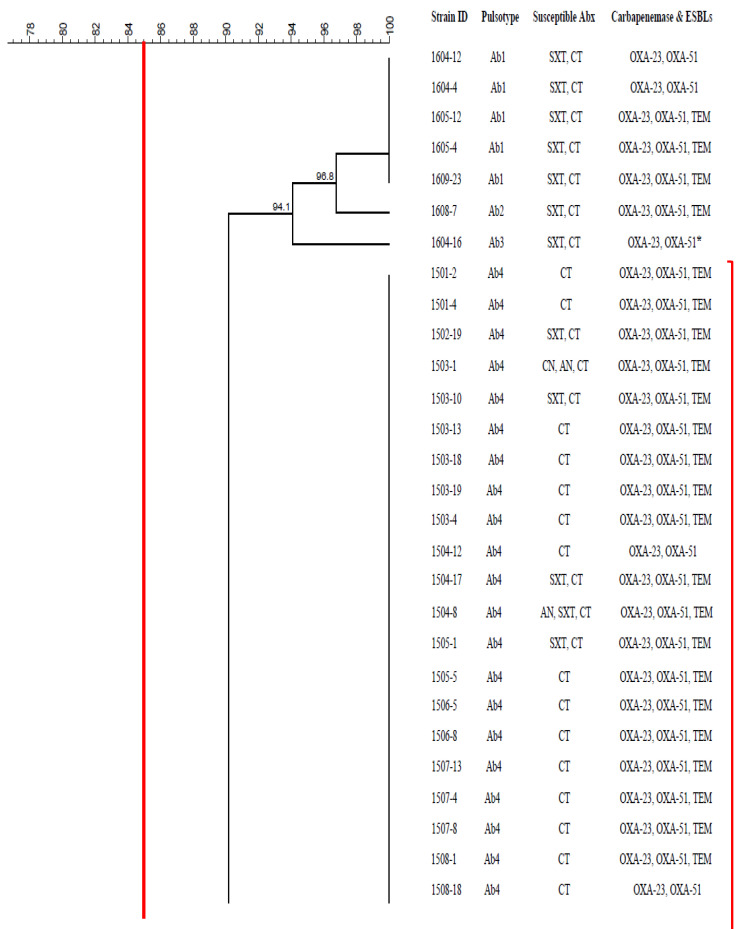

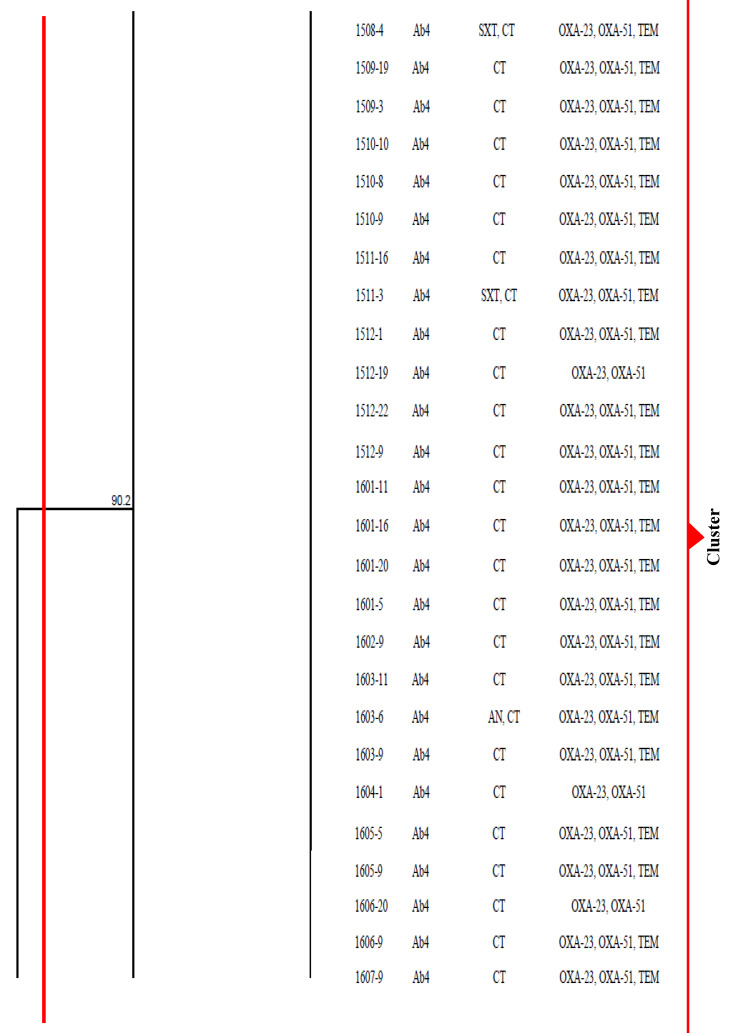

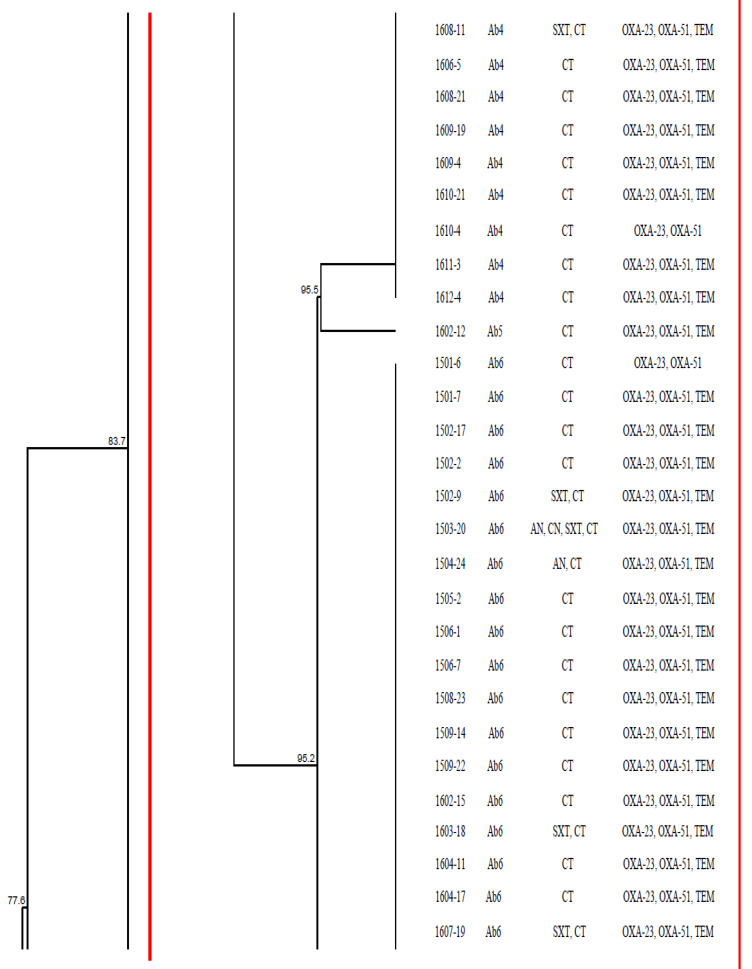

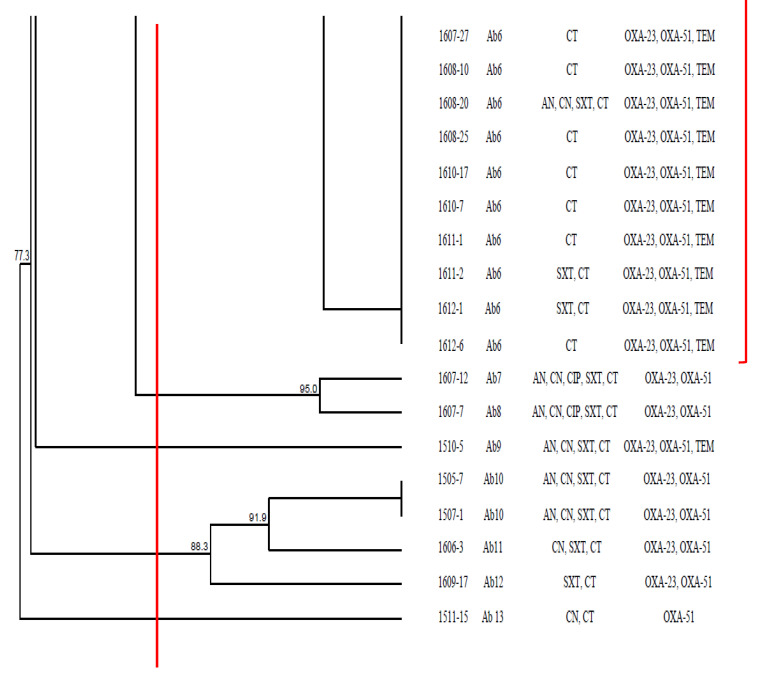
Dendrogram generated with dice coefficient and the UPGMA clustering method based on the PFGE banding pattern of 100 CRAB isolates, showing the genetic similarity among the isolates, together with their respective pulsotype, class of antibiotics in which the isolate remains susceptible to, the carriage of carbapenem and ESBL-resistant determinants. The red line represents the similarity threshold of 85%. Abx: Antibiotics; AN: Amikacin; CIP: Ciprofloxacin; CN: Gentamicin; SXT: Trimethoprim–sulfamethoxazole; CT: Colistin. *: Outgroup.

A PFGE gel image is shown below as a representative (Figure 2). Pulsed-field gel electrophoresis subtyped the 100 CRAB isolates into 13 distinct pulsotypes (Figure 3), with 21–23 restriction fragments. Each pulsotypes is arbitrarily designated from Ab 1 to Ab 13. Based on the dendrogram generated, using 85% similarity as a threshold level, 1 cluster was identified among the 100 isolates. This cluster consisted of 92 isolates, had 2 predominant pulsotypes, Ab 4 and Ab 6, accounting for 56% and 28% of the isolates, respectively.

### 2.6. Cox Proportional Hazards Regression Model for Risk Factors Analysis

**Figure 4:**
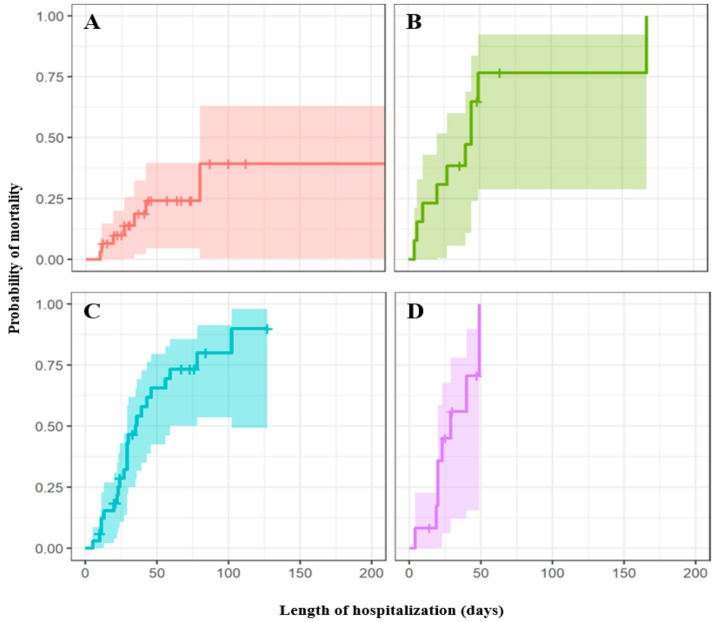
Kaplan–Meier survival curve that shows the relative probability of mortality based on Chinese ethnicity and diabetes mellitus (DM). (**A**–**D**) Patients are neither Chinese nor with DM (**A**), Chinese without DM (**B**), Non-Chinese with DM (**C**) and Chinese with DM (**D**), respectively.

The risk factors for patients’ mortality throughout the hospitalization were analysed by Cox proportional hazards model. Basically, two multivariate Cox regression models were created. In the first model (Table 2), risk factors of mortality among all 89 patients (regardless of infection or colonization) were evaluated, with the antibiotics data being excluded. In the univariate model, five variables including age groups, classification of isolates, ethnicity (Chinese), specimen source and comorbidity (no known medical illness, diabetes mellitus, hypertension, cardiovascular disease, brain and neurological disorder) were found to be significant (*p* value of < 0.15) confounding factors for patients’ in-hospital mortality. The abovementioned risk factors were selected to build the parsimonious model. In the multivariate Cox analysis, the combination of Chinese ethnicity and presence of diabetes mellitus were selected. Specifically, the analysis revealed that patients who are Chinese and patients with diabetes mellitus as an underlying disease will have a higher risk of mortality. Kaplan–Meier survival curves (Figure 4) were created to illustrate the survival time of patient with diabetes mellitus, patients who are Chinese or the combination of both. The survival curve showed that patients who met the mentioned criteria (Chinese ethnicity, diabetes mellitus or both) tended to have a greater risk of mortality during hospitalization.

In the second model (Table 3), risk factors for mortality were analysed again among patients infected with CRAB, with antibiotic data being included in the analysis. Variables including comorbidity (diabetes mellitus, malignancy, kidney disease, cardiovascular disease, brain and neurological disorder, blood and immune disease) and prescription of carbapenem as empiric treatment were found to be significant. Multivariate analysis showed that the presence of comorbidities significantly increases the mortality rate of patients who have CRAB infection.

## 3. Discussion

*A. baumannii* infection is now becoming increasingly difficult to treat due to the emergence of strains that are resistant to nearly all available antimicrobial agents. Infections caused by CRAB normally lead to prolonged hospitalization, increased costs of treatments and greater mortality as compared with carbapenem-susceptible strains [2]. Except for colistin, CRAB isolates tested in this study were extremely resistant to most clinically available antibiotics. Such a phenomenon is worrying, as limited therapeutic options are available for treatment against CRAB. The optimal treatment of infections due to this organism is contentious. In most cases, polymyxin/colistin monotherapy is used; however, combined therapy with tigecycline or sulbactam has been associated with high probability of success, although controversy remains regarding the most effective combination [19].

Even though colistin is effective, its association with nephrotoxicity cannot be neglected. A systematic review and meta-analysis conducted to evaluate the safety and efficacy of colistin alone or in combination in adults with CRAB infection showed that the administration of colistin alone in treatment group may contribute to the development of nephrotoxicity in 39.3% of the patients, which, in turn, puts patients at the risk of mortality [20]. To mitigate this issue, inhaled colistin therapy has been introduced and proven to increase microbial eradication rate while at the same time minimize the systemic toxicity. However, colistin is still toxic to the lung tissue and can induce bronchospasm in certain circumstances [2]. In our ICUs, the treatments against CRAB infection is colistin in combination with high dose ampicillin sulbactam [21]. Although new antibiotics such as cefiderocol and fluorocycline have been shown to pose good activity against CRAB, appropriate use of antibiotics and the continuous antimicrobial susceptibility profiling are needed to prevent the emergence of strains that show heteroresistance against these leftover antibiotic choices.

Carbapenem resistance in *A. baumannii* is mainly attributed to three major complementary mechanisms, including enzymatic inactivation, defects in porin and the action of efflux pumps [9]. However, the production of enzyme such as metallo-β-lactamases and oxacillinase remain as the most common and prevalent mechanisms. The CRAB isolates from UMMC expressed mainly OXA carbapenemase-mediated resistance, with 100% of the isolates harbouring at least one OXA-carbapenemase genes. The *bla*OXA-51-like gene is present in all isolates, supporting the understanding that this gene is intrinsic to *A. baumannii* species and can be used as a marker for identification [22]. Furthermore, 99% of the isolates coharboured *bla*OXA-23-like gene. The prevalence rate of 99% reported in the current study is in concordance with most studies reported in Malaysia and other Asian countries [23]. A systematic review conducted in Malaysian hospitals concluded that *bla*OXA-23-like gene is the predominant acquired carbapenemase in Malaysian *A. baumannii* isolates [8]. As of year 2011, the prevalence rate of *bla*OXA-23 in Hospital Sultanah Nur Zahirah and UKMMC was 75.9% and 82%, respectively. Other OXA carbapenemase genes including *bla*OXA-24-like and *bla*OXA-58-like were absent in our isolates. This finding is consistent with a parallel study from Malaysia and other Southeast Asian countries [8,23]. Although metallo-β-lactamases genes were absent in our study, these genes have been reported in other local clinical settings previously at relatively low prevalence. For instance, *bla*IMP has been reported with UKMMC of only 9.9% of the *A. baumannii* isolates and 5.1% of the carbapenem-resistant UMMC *Acinetobacter* spp. [8]. The high prevalence of OXA carbapenemases compared with carbapenemases from other classes may be attributed to two reasons. Firstly, due to the variability of its amino acid sequences, there are limited inhibitors against the OXA enzymes. Class A carbapenemase such as *bla*KPC tends to be inhibited by clavulanic acid, tazobactam, sulbactam and avibactam, whereas the activity of metallo-β-lactamases is easily compromised by EDTA and cefaclor [24]. Secondly, the ISA*ba1* insertion in the promoter region of OXA carbapenemase gene is capable of inducing OXA carbapenemase overexpression, which, in turn, allows CRAB to confer high-resistance to carbapenem antibiotic, even though OXA carbapenemase exhibits lower affinity towards carbapenem if compared with carbapenemase from other classes [25].

Notwithstanding to the activity of carbapenemase, the role of outer membrane protein, especially carbapenem-associated outer membrane protein (*Car*O), is found to be another contributing factor for carbapenem resistance [9]. There are limited studies concerning the impact of changes in the membrane protein towards antibiotic resistance, and until now, the exact role of *Car*O in carbapenem resistance has not been well elucidated [26]. It is believed that carbapenem resistance is due to the defects in outer membrane porin, which can be achieved either through the insertional inactivation of the gene or amino acid mutation leading to the reduced affinity of this protein towards carbapenem as well as the total loss of this outer membrane protein [27]. Separately, although the insertion sequence in *Car*O structure was reported previously, this phenomenon was absent in this study [28,29]. Nonetheless, the presence of indel along the nucleotide sequence has brought about some changes to the amino acid sequence. It has been reported that the changes in the primary structure of the *Car*O protein will impose a dramatic impact on the entry of imipenem into the cell, thus contributing to resistance to carbapenem [30]. Thus, a further study on the relative expression of this gene is needed to validate the association of *Car*O alleles with the resistance phenotype.

CRAB is one of the most problematic pathogens to contain, and a successful containment requires intensive, long-term infection control measures [19]. Achieving control and prevention of on pathogen dissemination requires their clonal relationships to be elucidated clearly [31,32]. In the present study, PFGE, which is still considered as a gold standard for bacteria typing in most developing countries, was used to perform CRAB molecular typing [33]. Owing to its high discriminatory power, cost affordability and technical relevance, such technique is widely employed for outbreak tracing in most of the developing countries. Comparison between PFGE profiles of all isolates in this study showed a low genetic diversity of CRAB in ICU. The presence of only one cluster with 92 isolates (Cluster 1) among 100 isolates demonstrated the clonal spread of this pathogen in this ICU. A similar study has been reported in a tertiary-care hospital in Johor, Malaysia, in which the authors reported the presence of dominant genotype among 74% of the isolates [34]. In UMMC, the last CRAB genotyping effort can be traced back to 7 years ago, where 98 pulsotypes with 8 clusters among 170 clinical isolates was identified using PFGE [35]. The high clonal diversity in that study indicated that the acquisition of CRAB in ICU was mainly stochastic, with no obvious cross transmission pattern detected. The transition from high to low clonal diversity, coupled with the emergence of Ab 4 and Ab 6, the predominant pulsotypes in our study suggested the competitive advantages of these pulsotypes against other pulsotypes. Indeed, most of the isolates from Cluster 1 in this study were extremely resistant to most antimicrobial agents, except for colistin, compared to those sporadic pulsotypes. Such findings may indicate that the possession of extremely resistant phenotype allows CRAB belonging to these two pulsotypes to survive and remain endemic in the ICU. It is also perhaps not surprising given that critically ill patients are normally being prescribed more than one type of antibiotics, which, in turn, creates selection pressure leading to the emergence of highly resistant strains [36]. The detection of closely related strains from most of the patients in this study suggests that the ICU itself serves as a reservoir for this pathogen. These isolates that showed 100% similarity represent an endemic CRAB clone causing ongoing outbreaks in the ICU. Its spread and persistence is difficult to control because this species can survive for prolonged periods on environmental surfaces and also human skin, especially in areas with hot and humid climate [37]. Cross transmission between patients from contaminated equipment (ventilators, infusion pump), poor adherence to infection control practices such as hand hygiene and contact isolation practices (appropriate use of personal protective equipment, isolation of patients, disinfection of equipment) may contribute to this persistence in ICU [38]. Further investigations such as active culture surveillance, strengthening infection control practices and the study of the strains’ persistence mechanisms such as biofilm forming ability, desiccation tolerance and biocides resistance are needed for us to understand the endemicity, as well as to eradicate this pathogen from the institution.

In the present study, factors including age groups, infection/ colonization, ethnicity, comorbidity and CRAB specimen source were found to be significant with patients’ mortality. A total of 54% of the patients died during their hospital stay. Among the non-survivors (*n* = 48), two-thirds of them belonged to the age group of more than 50 years old, suggesting that geriatric patients are at a higher risk of mortality. Similar findings have been reported in previous studies [39,40]. In terms of the infection/ colonization, 77% of the patients infected with CRAB passed away during hospital stay. Undoubtedly, patients with CRAB infection often experienced higher risk of mortality, compared to those infected by carbapenem-susceptible *A. baumannii* or CRAB colonization [41,42]. When analysing all these variables in combination, Cox multivariate analysis suggested that the ethnic Chinese and those with diabetes mellitus have a higher risk of mortality. In a recent study, hyperglycaemia, even with mild glucose elevation, is found to be associated with increased mortality in critically ill patients, regardless of the illness severity [43]. In this study, Chinese ethnicity was significantly associated with a higher risk of mortality. One plausible explanation for this finding could be that Chinese patients have a different host genetic factors that predisposes them to more severe disease outcomes compared to other ethnic groups. Though currently there are no similar reports of risk of mortality due to CRAB in those of Chinese ethnicity, a study in Singapore has shown that Chinese patients were infected by capsule type K1 hypervirulent variant of *Klebsiella pneumoniae* strains at a significantly higher frequency than other ethnics [44]. Hence, further research on host genetic factors is needed to ascertain the reason of this association.

In this study, the risk factors for mortality among patients infected with CRAB was further evaluated, and we found that patients’ comorbidities are the significant factor for mortality. Cardiovascular disease, malignancy, blood and immune disease and brain and neurological disorder tend to increase a patient’s risk of mortality. There are many reports showing that the prognosis of patients with CRAB infection is also dependant on the host condition, such as the severity of disease and immune status. Du et al. (2019), in their study, reported that the fatality rate of patients with underlying illnesses such as chronic liver and renal disease and hypertension was higher in different degrees than in patients without these conditions [4]. Similarly, Garnacho-Montero et al. and Sileem et al. showed that comorbidities are a high significant predictor for severity of CRAB infection [45,46].

There are several limitations in the present study. Firstly, not all the CRAB isolates in ICU from 2015–2016 were included in this study. Out of 134 isolates, 100 (75%) were selected. Nevertheless, these 100 isolates consist of 3 to 6 strains from each month as a representative to monitor the changing in their molecular pattern throughout the 2 years and to identify the ongoing clonal spread.

Secondly, among those 100 patients, only 89 patients’ medical record were retrieved successfully. Those with incomplete data were excluded from the analysis. Despite these shortcomings, the present study adds value to the knowledge gaps regarding the epidemiology data of *Acinetobacter baumannii* in Malaysia, as this is a more comprehensive study looking at both clinical and molecular factors of the pathogens. Findings from this study may assist in curbing the consistently high incidence and prevalence rate of CRAB in the clinical settings.

## 4. Materials and Methods

### 4.1. Ethical Clearance

Medical ethics in conducting research on clinical isolates and accessing the patients’ clinical data was approved by UMMC Medical Ethics Committee (MREC ID: 2019815-7748).

### 4.2. Study Setting, Bacterial Strain Collection and Identification

This retrospective study was conducted in general adult ICU in University of Malaya Medical Centre (UMMC), a tertiary teaching hospital located in Kuala Lumpur, Malaysia. The 25-bedded ICU had both surgical and medical cases and admitted 1319 and 2105 patients in 2015 and 2016, respectively. In 2015 and 2016, there were a total of 134 CRAB clinical isolates for the ICU. Each month, 3 to 6 isolates were selected, and a total of 100 isolates were obtained and included in the study. The strains collected were from various clinical specimen samples, including blood, urine, sputum, bronchoalveolar lavage, cerebrospinal fluid and others. Isolate identities were first confirmed using Vitek^®^ 2 system (BioMérieux, Marcy L’Etoile, France), followed by validation using duplex PCR targeting *rec*A gene and intergenic spacer region of 16S-23S rRNA [47].

### 4.3. Antimicrobial Susceptibility Testing

Susceptibility profiles of the isolates to antibiotics including ampicillin–sulbactam, piperacillin–tazobactam, ciprofloxacin, imipenem and meropenem were retrieved from the results of Vitek^®^ 2 system (BioMérieux, Marcy L’Etoile, France). Minimum inhibitory concentration (MIC) values of imipenem, meropenem and colistin were determined using the broth microdilution method, according to CLSI guidelines [48]. In brief, imipenem and meropenem resistance were defined as MIC ≥ 8 µg/mL, while colistin resistance was defined as MIC ≥ 4 µg/mL.

### 4.4. Carbapenemase and Extended-Spectrum β-Lactamase Genes Screening (ESBL)

Polymerase chain reaction (PCR) was used for detection of genes encoding ESBL such as *bla*_TEM_, *bla*_SHV_, *bla*_CTX-M_ and carbapenemase genes such as *bla*_NDM_, *bla*_VIM,_ and *bla*_IMP_ based on the primers and condition reported in other studies [49,50,51]. The presence of Class D carbapenemase genes (*bla*_OXA-23_, *bla*_OXA-24_, *bla*_OXA-51_, *bla*_OXA-58_) were screened by multiplex PCR based on the primers and conditions, as described previously [52]. The amplified products were sequenced and blasted against the NCBI nucleotide database for validation of identity.

### 4.5. CarO Gene Screening and Amino Acid Sequence Alignment

The presence of porin-associated gene, *Car*O, was screened by using the primers and thermocycling conditions described by Mussi et al. [29]. The deduced amino acid sequences obtained from all the *Car*O genes from this study were aligned with *Car*Oa (ATCC 19606) and *Car*Ob (A6 strain) using ClustalW to observe for any mutation in the porin.

### 4.6. Pulsed-Field Gel Electrophoresis (PFGE)

Genetic relatedness of the isolates was revealed using PFGE. Briefly, genomic DNA of CRAB contained in the plug was digested using *Apa*I restriction enzyme (Promega, Madison, WI, USA). The fragmented DNA was separated using PFGE CHEF-MAPPER system (Bio-Rad, Hercules, CA, USA) under the condition of 6 Vcm^−1^ for 20 h, with initial and final switch time of 2 s and 40 s, respectively [53]. *Salmonella enterica*, serotype Braenderup H9812, was used as the reference standard marker. Bionumerics software, version 7.6.2 (AppliedMaths, Sint–Martens–Latem, Belgium), was used for the analysis of banding patterns. A dendrogram was generated based on dice correlation coefficients and clustered by unweighted pair group method using arithmetic averages (UPGMA) with band position tolerance and optimization of 1.5% and 2.0%, respectively. Isolates grouped together based on > 85% similarity in dendrogram were considered to represent the same PFGE cluster.

### 4.7. Clinical Data Collection and Statistical Analysis

Patients’ demographics and clinical data including age, ethnic, gender, comorbidity, length of hospital and ICU stay, source of specimen, classification based on whether the isolates were due to colonization or infection, previous antibiotics exposure, empiric antibiotic therapy and patients’ outcome were retrieved from patients’ medical records. Infection was defined as isolates from patients who had evidence of infection based on the National Nosocomial Infections Surveillance criteria. Empiric antibiotic therapy is defined as the initial antibiotic administered in the 48 h after the onset of symptoms before the release of the microbiological results. Definitive treatment is defined as administrating of one (monotherapy) or more than one (combined therapy) active antibiotic against CRAB, which includes any antibiotic that is reported as sensitive in the microbiological results such as colistin/polymyxin and high-dose ampicillin–sulbactam. Administration of targeted therapy within and after 3 days of obtaining samples was also compared.

Risk factors associated with in-hospital all-cause mortality were first analysed using IBM SPSS ^TM^ Statistic software, version 25 (SPSS, Chicago, IL, USA). Categorical variables were compared using chi-square test, while continuous variables were analysed using Student’s t-test or Mann–Whitney U test, depending on the data normality. A *p*-value of <0.05 was considered statistically significant.

Cox proportional hazards model was used to evaluate the relationship between potential risk factors with the patients’ survival time. The univariate Cox regression analysis was performed with a survival package under R statistical software, version 3.5.2 (RStudio). Further stepwise variable selection was performed with “My.stepwise” R package to identify the parsimonious model. Significance levels for entry (SLE) and stay (SLS) were set at 0.150, in which predictors with *p* < 0.150 were included in the stepwise selection. A forest plot for Cox proportional hazards model was generated, and the Kaplan–Meier survival curve were used for survival analysis. Separately, the Cox proportional hazards model was generated again to evaluate the potential risk factors of mortality among the patients infected with CRAB.

## 5. Conclusions

Rapid emergence of CRAB in healthcare settings imposes a great threat for patients’ well-being, and the dissemination of strains with extremely resistant phenotypes will inevitably compromise the treatment of CRAB infection in vulnerable groups. While multi- and pan-drug-resistant *A. baumannii* becomes more and more prevalent, available options for treatment are declining. The present study revealed the circulation and endemicity of extremely drug-resistant CRAB in adult ICUs. Without proper interventions, any lapse in the infection control measures will bring about the occurrence of outbreak in ICUs. Hence, early identification of the source; implementing comprehensive infection control measures, which include improving hand hygiene, standard precaution, contact precaution and environment and equipment disinfection; and education, training, audit and feedback are of utmost importance to halt the dissemination of CRAB in ICUs. Findings obtained from this study can be used as a baseline knowledge for improving patients’ clinical outcome, and, at the same time, serves as useful data for clinicians to review the antibiotic stewardship in the clinical settings.

## Figures and Tables

**Table 1:** Univariate analysis of the clinical characteristics of patients with CRAB who survived and did not survive.

Risk Factors	In-Hospital Mortality		*p*-Value ^1^
	Survivor (*n* = 41)	Non-Survivor (*n* = 48)	
**Gender**			0.309
Male	29 (50.0%)	29 (50.0%)	
Female	12 (38.7%)	19 (61.3%)	
**Classification of isolates**			**0.006 ^2^**
Colonization	21 (65.5%)	11 (34.5%)	
Infection	20 (35.1%)	37 (64.9%)	
**Age**			**0.003**
≤50 years old	23 (65.7%)	12 (34.3%)	
>50 years old	18 (33.3%)	36 (66.7%)	
**Septic shock during CRAB isolation**			0.501
Yes	21 (42.9%)	28 (57.1%)	
No	20 (50.0%)	20 (50.0%)	
**Ethnicity**			
Chinese	7 (28.0%)	18 (72.0%)	**0.033**
Malay	12 (44.4%)	15 (55.6%)	0.839
Indian	14 (48.3%)	15 (51.7%)	0.771
Others	8 (100%)	0	**0.001**
**Specimen source**			
Respiratory	29 (49.2%)	30 (50.8%)	0.413
Urinary	3 (75.0%)	1 (25.0%)	0.235
Blood	5 (31.3%)	11 (68.7%)	0.189
Skin and soft tissue	4 (57.1%)	3 (42.9%)	0.540
Others	0	3 (100%)	0.103
**Comorbidity**			
No known medical illness	15 (68.2%)	7 (31.8%)	**0.016**
Diabetes mellitus	14 (31.1%)	31 (68.9%)	**0.004**
Hypertension	19 (37.3%)	32 (62.7%)	0.053
Malignancy	3 (27.3%)	8 (72.7%)	0.182
Kidney disease	9 (39.1%)	14 (60.9%)	0.438
Cardiovascular disease	3 (18.8%)	13 (81.2%)	**0.015**
Endocrine and metabolic disorder	6 (42.9%)	8 (57.1%)	0.793
Respiratory disease	2 (20.0%)	8 (80.0%)	0.079
Skin disease	1 (100%)	0	0.277
Bone and joint disease	1 (50.0%)	1 (50.0%)	0.910
Brain and neurological disorder	1 (20.0%)	4 (80.0%)	0.229
Liver and pancreas disease	1 (50.0%)	1 (50.0%)	0.910
Gastrointestinal disease	1 (100%)	0	0.277
Blood and immune disease	2 (50.0%)	2 (50.0%)	0.872
**Empiric treatment (*n* = 57)**			
Beta-lactamase/Beta-lactamase inhibitor	10 (41.7%)	14 (58.3%)	0.375
Tetracycline	1 (100%)	0	0.170
Trimethoprim–sulfamethoxazole	1 (33.3%)	2 (66.7%)	0.948
Carbapenem	8 (23.5%)	26 (76.5%)	**0.026**
Colistin	0	4 (100%)	0.127
**Definitive treatment (*n* = 57)**			0.700
Within 3 days	2 (28.6%)	5 (71.4%)	
More than 3 days	18 (36.0%)	32 (64.0%)	
**Length of hospitalization stay**	51.66 ± 38.317	33.65 ± 28.312	**0.003**
**Length of ICU stay**	20.10 ± 15.043	21.06 ± 16.015	0.699

^1^*p* value is obtained based on the univariate analysis using SPSS software. ^2^ Bold text indicates *p* value of < 0.05, which is statistically significant.

**Table 2:** Cox proportional hazard model for patients’ in-hospital all-cause mortality associated with carbapenem-resistant *Acinetobacter baumannii* (*n* = 89).

Variables	Univariate Analysis			Multivariate Analysis		
	Hazard Ratio (HR)	95% CI for HR	*p* Value	Odds Ratio	95% CI for HR	*p* Value
**Gender**	0.94	0.52–1.70	0.83			
**Classification of isolates**	1.60	0.83–3.20	0.15			
**Age group**	0.45	0.23–0.86	0.016			
**Septic shock during CRAB isolation**	1.40	0.80–2.50	0.24			
**Ethnic**						
**Chinese**	1.90	1.10–3.50	0.032	2.13	1.17–3.89	0.014
**Malay**	1.10	0.57–2.00	0.85			
**Indian**	0.83	0.44–1.50	0.55			
**Others**	1.30 × 10^−8^	0.00–lnF	1.00			
**Specimen source**	1.20	0.95–1.50	0.12			
**Comorbidity**						
**No known medical illness**	0.46	0.21–1.00	0.06			
**Diabetes mellitus**	2.40	1.30–4.40	0.0045	2.59	1.40–4.78	0.002
**Hypertension**	1.60	0.88–2.90	0.12			
**Malignancy**	1.70	0.78–3.60	0.19			
**Kidney disease**	1.40	0.75–2.60	0.30			
**Cardiovascular disease**	2.30	1.20–4.30	0.013			
**Endocrine and metabolic disorder**	0.98	0.46–2.10	0.96			
**Respiratory disease**	1.40	0.63–3.00	0.43			
**Skin disease**	3.00 × 10^−7^	0.00–lnF	1.00			
**Bone and joint disease**	0.80	0.11–5.80	0.82			
**Brain and neurological disorder**	2.30	0.83–6.60	0.11			
**Liver and pancreatic disease**	1.50	0.21–11.0	0.68			
**Gastrointestinal disease**	1.10 ×10^−7^	0.00–lnF ^1^	1.00			
**Blood and immune disease**	1.10	0.26–4.50	0.92			

^1^ lnF: Infinity.

**Table 3:** Cox proportional hazard model for in-hospital all-cause mortality associated with CRAB infection (*n* = 57).

Variables	Univariate Analysis			Multivariate Analysis		
	Hazard Ratio (HR)	95% CI for HR	*p* Value	Odds Ratio	95% CI for HR	*p* Value
**Gender**	0.98	0.50–1.90	0.94			
**Age group**	0.78	0.37–1.60	0.51			
**Septic shock during CRAB isolation**	1.50	0.74–3.00	0.27			
**Ethnic**						
**Chinese**	1.60	0.82–3.20	0.17			
**Malay**	0.93	0.46–1.90	0.83			
**Indian**	0.91	0.44–1.90	0.79			
**Others**	1.30 × 10^−8^	0.00–lnF	1.00			
**Specimen source**	1.10	0.89–1.40	0.30			
**Comorbidity**						
**No known medical illness**	0.52	0.20–1.30	0.18			
**Diabetes mellitus**	1.80	0.91–3.70	0.091			
**Hypertension**	1.50	0.75–3.00	0.25			
**Malignancy**	2.40	0.97–6.10	0.057	3.297	1.098–9.897	0.0334
**Kidney disease**	1.70	0.86–3.50	0.12			
**Cardiovascular disease**	3.50	1.50–8.20	0.003	5.804	2.315–14.553	0.0002
**Endocrine and metabolic disorder**	1.10	0.44–2.60	0.88			
**Respiratory disease**	1.10	0.46–2.70	0.81			
**Skin disease**	8.10 × 10^−7^	0.00–lnF ^1^	1.00			
**Bone and joint disease**	1.50	0.20–11.0	0.71			
**Brain and neurological disorder**	17.0	3.30–89.0	0.00072	42.614	7.34–247.406	0.00003
**Liver and pancreatic disease**	3.80	0.50–29.0	0.20			
**Blood and immune disease**	7.60	1.60–35.0	0.01	7.232	1.237–41.089	0.0256
**Empiric treatment**						
**Β-lactam/Β-lactam inhibitor**	0.61	0.30–1.20	0.17			
**Tetracycline**	3.90 × 10^−8^	0.00–lnF	1.00			
**Trimethoprim–sulfamethoxazole**	1.10 ×10^−8^	0.00–lnF	1.00			
**Carbapenem**	1.80	0.85–3.70	0.13			
**Colistin**	1.90	0.67–5.60	0.22			
**Appropriate treatment with 3 days**	1.10	0.41–2.80	0.89			

^1^ lnF: Infinity.

## References

[1] Brink A.J. (2019). Epidemiology of carbapenem-resistant Gram-negative infections globally. Curr. Opin. Infect. Dis..

[2] Wong D., Nielsen T.B., Bonomo R.A., Pantapalangkoor P., Luna B., Spellberg B. (2017). Clinical and pathophysiological overview of *Acinetobacter* infections: A century of challenges. Clin. Microbiol. Rev..

[3] Liang C.A., Lin Y.C., Lu P.L., Chen H.C., Chang H.L., Sheu C.C. (2018). Antibiotic strategies and clinical outcomes in critically ill patients with pneumonia caused by carbapenem-resistant *Acinetobacter baumannii*. Clin. Microbiol. Infect..

[4] Du X., Xu X., Yao J., Deng K., Chen S., Shen Z., Yang L., Feng G. (2019). Predictors of mortality in patients infected with carbapenem-resistant *Acinetobacter baumannii*: A systematic review and meta-analysis. Am. J. Infect. Control.

[5] Piperaki E.T., Tzouvelekis L., Miriagou V., Daikos G. (2019). Carbapenem-resistant *Acinetobacter baumannii*: In pursuit of an effective treatment. Clin. Microbiol. Infect..

[6] Shrivastava S.R., Shrivastava P.S., Ramasamy J. (2018). World health organization releases global priority list of antibiotic-resistant bacteria to guide research, discovery, and development of new antibiotics. J. Medic. Soc..

[7] Gonzalez-Villoria A.M., Valverde-Garduno V. (2016). Antibiotic-resistant *Acinetobacter baumannii* increasing success remains a challenge as a nosocomial pathogen. J. Pathog..

[8] Rahman A., Iza N., Ismail S., Alattraqchi A.G., Cleary D.W., Clarke S.C., Yeo C.C. (2017). *Acinetobacter* spp. infections in Malaysia: A review of antimicrobial resistance trends, mechanisms and epidemiology. Front. Microbiol..

[9] Peleg A.Y., Seifert H., Paterson D.L. (2008). *Acinetobacter baumannii*: Emergence of a successful pathogen. Clin. Microbiol. Rev..

[10] Vijayakumar S., Mathur P., Kapil A., Das B.K., Ray P., Gautam V., Sistla S. (2019). Molecular characterization & epidemiology of carbapenem-resistant *Acinetobacter baumannii* collected across India. Indian J. Med. Res..

[11] Zowawi H.M., Sartor A.L., Sidjabat H.E., Balkhy H.H., Walsh T.R., Al Johani S.M., Al Jindan R.Y., Alfaresi M., Ibrahim E., Al-Jardani A. (2015). Molecular epidemiology of carbapenem-resistant *Acinetobacter baumannii* isolates in the Gulf Cooperation Council States: Dominance of OXA-23-type producers. J. Clin. Microbiol..

[12] Li Y., Guo Q., Wang P., Zhu D., Ye X., Wu S. (2015). Wang, M. Clonal dissemination of extensively drug-resistant *Acinetobacter baumannii* producing an OXA-23 β-lactamase at a teaching hospital in Shanghai, China. J. Microbiol. Immunol..

[13] Wieland K., Chhatwal P., Vonberg R.P. (2018). Nosocomial outbreaks caused by *Acinetobacter baumannii* and *Pseudomonas aeruginosa*: Results of a systematic review. Am. J. Infect. Control.

[14] Saharman Y.R., Karuniawati A., Sedono R., Aditianingsih D., Sudarmono P., Goessens W.H., Klaassen C.H., Verbrugh H.A., Severin J.A. (2018). Endemic carbapenem-nonsusceptible *Acinetobacter baumannii-calcoaceticus* complex in intensive care units of the national referral hospital in Jakarta, Indonesia. Antimicrob. Resist. Infect. Control.

[15] Schultz M.B., Thanh D.P., Do Hoan N.T., Wick R.R., Ingle D.J., Hawkey J., Edwards D.J., Kenyon J.J., Lan N.P.H., Campbell J.I. (2016). Repeated local emergence of carbapenem-resistant *Acinetobacter baumannii* in a single hospital ward. Microb. Genom..

[16] Ning N.Z., Liu X., Bao C.M., Chen S.M., Cui E.B., Huang J., Chen F.H., Li T., Qu F., Wang H. (2017). Molecular epidemiology of bla OXA-23-producing carbapenem-resistant *Acinetobacter baumannii* in a single institution over a 65-month period in north China. BMC. Infect. Dis..

[17] Lean S.S., Yeo C.C., Suhaili Z., Thong K.L. (2015). Whole-genome analysis of an extensively drug-resistant clinical isolate of *Acinetobacter baumannii* AC12: Insights into the mechanisms of resistance of an ST195 clone from Malaysia. Int. J. Antimicrob. Agents.

[18] Lean S.S., Yeo C.C., Suhaili Z., Thong K.L. (2016). Comparative genomics of two ST 195 carbapenem-resistant *Acinetobacter baumannii* with different susceptibility to polymyxin revealed underlying resistance mechanism. Front. Microbiol..

[19] Weinberg S., Villedieu A., Bagdasarian N., Karah N., Teare L., Elamin W. (2020). Control and management of multidrug resistant *Acinetobacter baumannii*: A review of the evidence and proposal of novel approaches. Infect. Prev. Pract..

[20] Wang J., Niu H., Wang R., Cai Y. (2019). Safety and efficacy of colistin alone or in combination in adults with *Acinetobacter baumannii* infection: A systematic review and meta-analysis. Int. J. Antimicrob. Agents.

[21] University of Malaya Medical Centre UMMC On-Line Antibiotic Guideline 2020. https://farmasi.ummc.edu.my/ummc-on-line-antibiotic-guideline.

[22] Takebayashi Y., Findlay J., Heesom K.J., Warburton P.J., Avison M.B., Evans B.A. (2021). Variability in carbapenemase activity of intrinsic OxaAb (OXA-51-like) β-lactamase enzymes in *Acinetobacter baumannii*. J. Antimicrob. Chemother..

[23] Hsu L.Y., Apisarnthanarak A., Khan E., Suwantarat N., Ghafur A., Tambyah P.A. (2017). Carbapenem-resistant *Acinetobacter baumannii* and *Enterobacteriaceae* in south and southeast Asia. Clin. Microbiol. Rev..

[24] Aghamali M., Bialvaei A.Z., Aghazadeh M., Asgharzadeh M., Kafil H.S. (2017). Carbapenemase inhibitors. Rev. Med. Microbiol..

[25] Wong M.H.Y., Chan B.K.W., Chan E.W.C., Chen S. (2019). Over-expression of ISAba1-linked intrinsic and exogenously acquired OXA type carbapenem-hydrolyzing-class D-ß-lactamase-encoding genes is key mechanism underlying carbapenem resistance in *Acinetobacter baumannii*. Front. Microbiol..

[26] Sen B., Joshi S. (2016). Studies on *Acinetobacter baumannii* involving multiple mechanisms of carbapenem resistance. J. Appl. Microbiol..

[27] Catel-Ferreira M., Coadou G., Molle V., Mugnier P., Nordmann P., Siroy A., Jouenne T., De E. (2011). Structure–function relationships of CarO, the carbapenem resistance-associated outer membrane protein of *Acinetobacter baumannii*. J. Antimicrob. Chemother..

[28] Lu P.L., Doumith M., Livermore D.M., Chen T.P., Woodford N. (2009). Diversity of carbapenem resistance mechanisms in *Acinetobacter baumannii* from a Taiwan hospital: Spread of plasmid-borne OXA-72 carbapenemase. J. Antimicrob. Chemother..

[29] Mussi M.A., Limansky A.S., Viale A.M. (2005). Acquisition of resistance to carbapenems in multidrug-resistant clinical strains of *Acinetobacter baumannii*: Natural insertional inactivation of a gene encoding a member of a novel family of β-barrel outer membrane proteins. Antimicrob. Agents. Chemother..

[30] Fonseca E.L., Scheidegger E., Freitas F.S., Cipriano R., Vicente A.C.P. (2013). Carbapenem-resistant *Acinetobacter baumannii* from Brazil: Role of car O alleles expression and bla OXA-23 gene. BMC Microbiol..

[31] Metan G., Zarakolu P., Otlu B., Tekin I., Aytaç H., Bölek E.Ç., Metin B.C., Arsava E.M., Unal S. (2020). Emergence of colistin and carbapenem-resistant *Acinetobacter calcoaceticus-Acinetobacter baumannii* (CCR-Acb) complex in a neurological intensive care unit followed by successful control of the outbreak. J. Infect. Public. Health.

[32] Al-Hamad A., Pal T., Leskafi H., Abbas H., Hejles H., Alsubikhy F., Darwish D., Ghazawi A., Sonnevend A. (2020). Molecular characterization of clinical and environmental carbapenem resistant *Acinetobacter baumannii* isolates in a hospital of the Eastern Region of Saudi Arabia. J. Infect. Public Health.

[33] Neoh H.M., Tan X.E., Sapri H.F., Tan T.L. (2019). Pulsed-field gel electrophoresis (PFGE): A review of the “gold standard” for bacteria typing and current alternatives. Infect. Genet. Evol..

[34] Dhanoa A., Rajasekaram G., Lean S.S., Cheong Y.M., Thong K.L. (2015). Endemicity of *Acinetobacter calcoaceticus-baumannii* complex in an intensive care unit in Malaysia. J. Pathog..

[35] Kong B.H., Hanifah Y.A., Yusof M.Y.M., Thong K.L. (2011). Antimicrobial susceptibility profiling and genomic diversity of multidrug-resistant *Acinetobacter baumannii* isolates from a teaching hospital in Malaysia. Jpn. J. Infect. Dis..

[36] Timsit J.F., Bassetti M., Cremer O., Daikos G., De Waele J., Kallil A., Kipnis E., Kollef M., Laupland K., Paiva J.A. (2019). Rationalizing antimicrobial therapy in the ICU: A narrative review. Intens. Care. Med..

[37] Suranadi I.W., Fatmawati N., Aryabiantara I.W., Sinardja C.D., Saputra D.J., Senapathi T.G.A., Widnyana I.M.G., Nada I.K.W., Astuti M.K., Kumara V. (2019). *Acinetobacter baumannii* is an opportunistic pathogen as an MDRO in ICU. Bali. J. Anesthesiol..

[38] Ababneh Q., Abulaila S., Jaradat Z. (2021). Isolation of extensively drug resistant *Acinetobacter baumannii* from environmental surfaces inside intensive care units. Am. J. Infect. Control.

[39] Hassan W.M.N.W., Puzizer M.S., Deris Z.Z., Zaini R.H.M. (2020). Carbapenem resistant *Acinetobacter* Species infection in intensive care unit: The outcome and risk factors of mortality. Bangladesh. J. Med. Sci..

[40] Zhou H., Yao Y., Zhu B., Ren D., Yang Q., Fu Y., Yu Y., Zhou J. (2019). Risk factors for acquisition and mortality of multidrug-resistant *Acinetobacter baumannii* bacteremia: A retrospective study from a Chinese hospital. Medicine.

[41] Lemos E., de La Hoz F., Einarson T., McGhan W., Quevedo E., Castañeda C., Kawai K. (2014). Carbapenem resistance and mortality in patients with *Acinetobacter baumannii* infection: Systematic review and meta-analysis. Clin. Microbiol. Infect..

[42] Karakonstantis S., Gikas A., Astrinaki E., Kritsotakis E.I. (2020). Excess mortality due to pandrug-resistant *Acinetobacter baumannii* infections in hospitalized patients. J. Hosp. Infect..

[43] Falciglia M., Freyberg R.W., Almenoff P.L., D’Alessio D.A., Render M.L. (2009). Hyperglycemia-related mortality in critically ill patients varies with admission diagnosis. Crit. Care. Med..

[44] Lee I.R., Molton J.S., Wyres K.L., Gorrie C., Wong J., Hoh C.H., Teo J., Kalimuddin S., Lye D.C., Archuleta S. (2016). Differential host susceptibility and bacterial virulence factors driving *Klebsiella* liver abscess in an ethnically diverse population. Sci. Rep..

[45] Garnacho-Montero J., Amaya-Villar R. (2010). Multiresistant *Acinetobacter baumannii* infections: Epidemiology and management. Curr. Opin. Infect. Dis..

[46] Sileem A.E., Said A.M., Meleha M.S. (2017). *Acinetobacter baumannii* in ICU patients: A prospective study highlighting their incidence, antibiotic sensitivity pattern and impact on ICU stay and mortality. Egypt. J. Chest. Dis. Tuberc..

[47] Chen T.L., Siu L.K., Wu R.C., Shaio M.F., Huang L.Y., Fung C.P., Lee C.M., Cho W.L. (2007). Comparison of one-tube multiplex PCR, automated ribotyping and intergenic spacer (ITS) sequencing for rapid identification of *Acinetobacter baumannii*. Clin. Microbiol. Infect..

[48] CLSI (2017). Performance Standards for Antimicrobial Susceptibility Testing.

[49] Alyamani E.J., Khiyami M.A., Booq R.Y., Alnafjan B.M., Altammami M.A., Bahwerth F.S. (2015). Molecular characterization of extended-spectrum beta-lactamases (ESBLs) produced by clinical isolates of *Acinetobacter baumannii* in Saudi Arabia. Ann. Clin. Microbiol. Antimicrob..

[50] Shoja S., Moosavian M., Rostami S., Abbasi F., Tabatabaiefar M.A., Peymani A. (2016). Characterization of Oxacillinase and Metallo-β-Lactamas genes and molecular typing of clinical isolates of *Acinetobacter baumannii* in Ahvaz, south-west of Iran. Jundishapur. J. Microb..

[51] Poirel L., Walsh T.R., Cuvillier V., Nordmann P. (2011). Multiplex PCR for detection of acquired carbapenemase genes. Diagn. Microbiol. Infect. Dis..

[52] Woodford N., Ellington M.J., Coelho J.M., Turton J.F., Ward M.E., Brown S., Amyes S.G., Livermore D.M. (2006). Multiplex PCR for genes encoding prevalent OXA carbapenemases in *Acinetobacter* spp.. Int. J. Antimicrob. Agents.

[53] Seifert H., Dolzani L., Bressan R., van der Reijden T., van Strijen B., Stefanik D., Heersma H., Dijkshoorn L. (2005). Standardization and interlaboratory reproducibility assessment of pulsed-field gel electrophoresis-generated fingerprints of *Acinetobacter baumannii*. J. Clin. Microbiol..

